# Elephant trunk tip musculature reflects species differences in grasping behavior

**DOI:** 10.1038/s42003-025-08998-6

**Published:** 2025-12-08

**Authors:** Lennart Eigen, Pius Ladenburger, Blaž Brence, Ani Shubitidze, Daniel Baum, Thomas Hildebrandt, Michael Brecht

**Affiliations:** 1 Humboldt University Berlin, Bernstein Center for Computational Neuroscience Berlin, Philippstr. 13, Haus 6, Berlin, Germany; 2 Zuse Institute Berlin, Takustr. 7, Berlin, Germany; 3 Leibniz Institute for Zoo and Wildlife Research, Alfred-Kowalke-Str. 17, Berlin, Germany; 4 NeuroCure Cluster of Excellence, Humboldt University Berlin, Berlin, Germany

**Keywords:** Animal physiology, Phylogenetics

## Abstract

Elephants use their trunks, muscular hydrostats, to perform a plethora of tasks. Trunk tip morphology as well as grasping behavior differ between elephant species. While African savanna elephants (*Loxodonta africana*) use their dorsal and ventral finger for pinching movements, Asian elephants (*Elephas maximus*) prefer to wrap around objects with their one dorsal finger and ventral bulb trunk tip lip. Moreover, *E. maximus* can flip their ventral bulb backwards to clamp objects behind the trunk tip. Whether trunk tip musculature differs between elephant species and muscle architecture is reflected by preferred grasping behavior is, however, not clear. In this study, we performed dense muscle fascicle reconstruction of three *L. africana* and three *E. maximus* hemi-trunk tips using a combination of manual and automated segmentation of high-resolution microfocus tomography (microCT) scans. We distinguish three types of muscle fascicles: longitudinal (bending and shortening), radial (elongating) and transversal muscle fascicles (elongating). We found that trunk tips of *L. africana* consist to one third of longitudinal and two thirds radial/transversal muscle fascicles, likely aiding in their grasping behavior, while *E. maximus* trunk tips consist to two thirds of longitudinal and one third radial/transversal muscle fascicles, which is advantageous for their wrapping and backward clamping behavior.

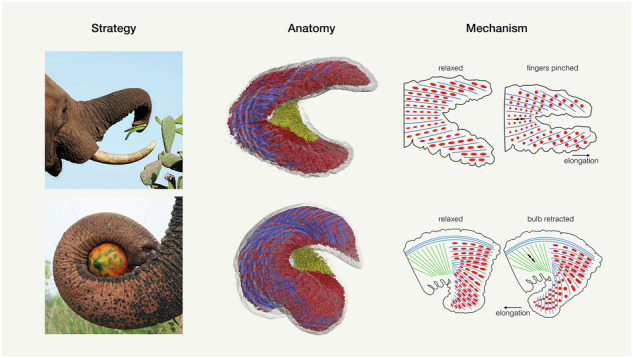

## Abstract (web summary)

High-resolution imaging reveals distinct muscle fascicle compositions in African and Asian elephant trunks, reflecting their preferred grasping modes and ecological niches.

## Introduction

The elephant trunk is a fascinating facial specialization. Elephants use it to pick up objects of up to 60 kg^1^, such as fruit from the floor and leaves from trees, or to suck up water that they then spray into their mouth^2^. It has elaborate sensory capacities and receives massive sensory tactile innervation^3^ with complex peripheral tactile specializations^4^, which allows for very fine-tuned movements. While the trunk musculature of all extant elephant species is similar^5^, they differ in their trunk tip morphology and grasping behavior. African savanna (*Loxondonta africana*) and African bush (*L. cyclotis*) elephant trunk tips have a dorsal and ventral finger and grasp objects by a pinching movement^6^. On the other hand, Asian elephant (*Elephas maximus*) trunk tips have a dorsal finger and thickened ventral bulb, which they use to wrap around objects^6^. In our study, we used *L. africana* and *E. maximus*. Due to inaccessibility, we did not include specimens of *L. cyclotis*. Like the arm of octopuses^7–12^ and tongue of mammals^10,13^, the elephant trunk is a muscular hydrostat^14–16^, it achieves movement independently of skeletal structures by muscles using each other for leverage, which allows an elephant to move its trunk in almost infinite degrees of freedom^7,8,15^.

In a recent study, Longren et al. ^14^ reconstructed muscle fascicles in the trunk of an *E. maximus* calf and estimated the number of muscle fascicles at 90,000. They propose that the tiny muscle fascicles in the trunk tip finger are one of the reasons for the elephants ability to do such dexterous movements. While we acknowledge ontogenetic variations in muscle size, we think that the general muscle architecture and relative size of muscle fascicles does not differ substantially between juvenile and adult elephants.

Three muscle fascicle types make up the trunk tip: longitudinal, radial, and transversal. Longitudinal muscle fascicles make up the outer layer of the musculature and run along the long axis of the trunk in both species. They are the longest muscle fascicles in the trunk and are presumably extensions of musculus maxillo-labialis^5^. When longitudinal muscle fascicles contract, the trunk gets shortened, allowing elephants to bend their trunk up-, down- and sidewards. Radial muscle fascicles are shorter and oriented orthogonally around the nostrils. They make up the majority of muscle fascicles in the trunk and are extraordinarily small in the most distal part of the trunk finger, which allows for very fine motor control^14^. Transversal muscle fascicles run orthogonally between the nostrils and are responsible for nasal contraction and dilation. They are smallest in the medial region and get larger towards the ventral and dorsal region. Both, radial and transversal muscle fascicles presumably are extensions of musculus rectus nasi and allow for an elongation of the trunk as well as an increase of the nostril volume^5^. It is, however, the complex interplay of all muscle fascicles, interwoven to form a dense, cross-hatched structure, that allows elephants to move their trunks in almost any direction.

In this study, we investigated the muscle architecture of *L. africana* and *E. maximus* trunk tips. We performed high-resolution microfocus computed tomography (microCT) scans of trunk tips of three adult *L. africana* and three adult *E. maximus* specimens. We then used a combination of manual and automated segmentation and classification algorithms to virtually dissect and classify the thousands of muscle fascicles. We asked following questions: (i) How does muscle architecture in *L. africana* and *E. maximus* trunk tips differ? (ii) What is the ratio of different muscle fascicle types in *L. africana* and *E. maximus* trunk tips? (iii) Is the preferred mode of grasping reflected in trunk tip muscle architecture?

## Results

### Elephant trunk tips and grasping strategies

**Fig. 1:**
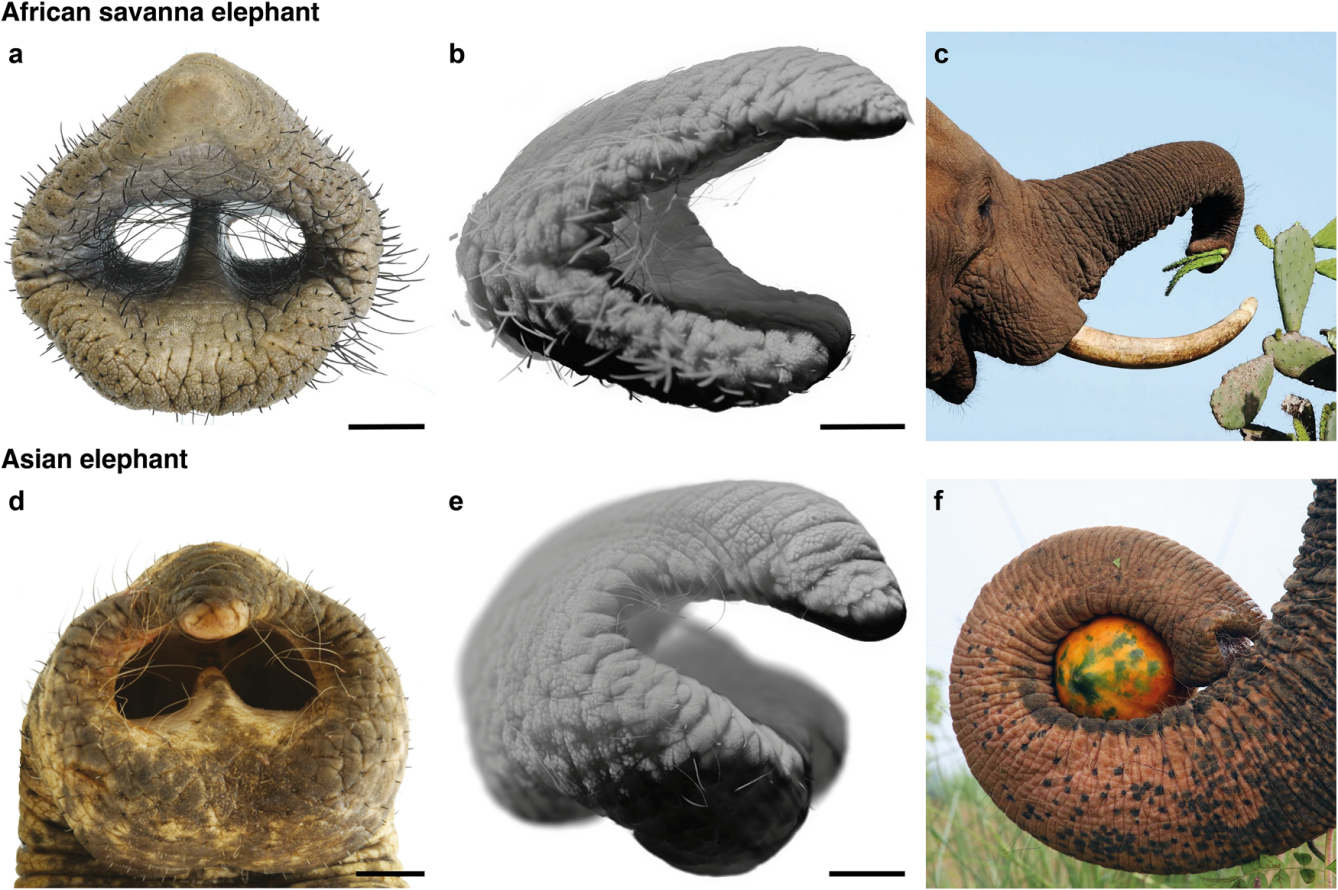
Trunk tips and grasping strategies of African savanna (*Loxodonta africana*) and Asian elephants (*Elephas maximus*). **a** Frontal view of *L. africana* trunk tip (Indra). **b** Lateral view of volume rendering of *L. africana* trunk tip from microCT scan (Indra). **c** Pinching strategy in *L. africana*. Elephant grasps cactus with its two trunk fingers. **d** Frontal view of *E. maximus* trunk tip (Vilja). **e** Lateral view of volume rendering of *E. maximus* trunk tip from microCT scan (Schoepfi). **f** Wrapping strategy in *E. maximus*. Elephant wraps around papaya with ventral bulb. Credit: Wildlife SOS/Mradul Pathak. Scale bars (**a**, **b**, **d**, **e**), 20 mm.

The trunk tip of *L. africana* has two triangular protrusions, the so-called dorsal and ventral trunk fingers (Fig. 1a, b). As first noted by Racine^6^, *L. africana* tends to pinch food items with its two trunk fingers (Fig. 1c). The trunk tip of *E. maximus*, however, only has one dorsal finger and a thickened ventral bulb (Fig. 1d, e). They use their bulb for clamping and wrapping around objects (Fig. 1f; Reference 5). Our study explores the muscular underpinnings of the different grasping strategies of these two elephant species.

### Muscle fascicle types in African savanna and Asian elephant trunk tips

**Fig. 2:**
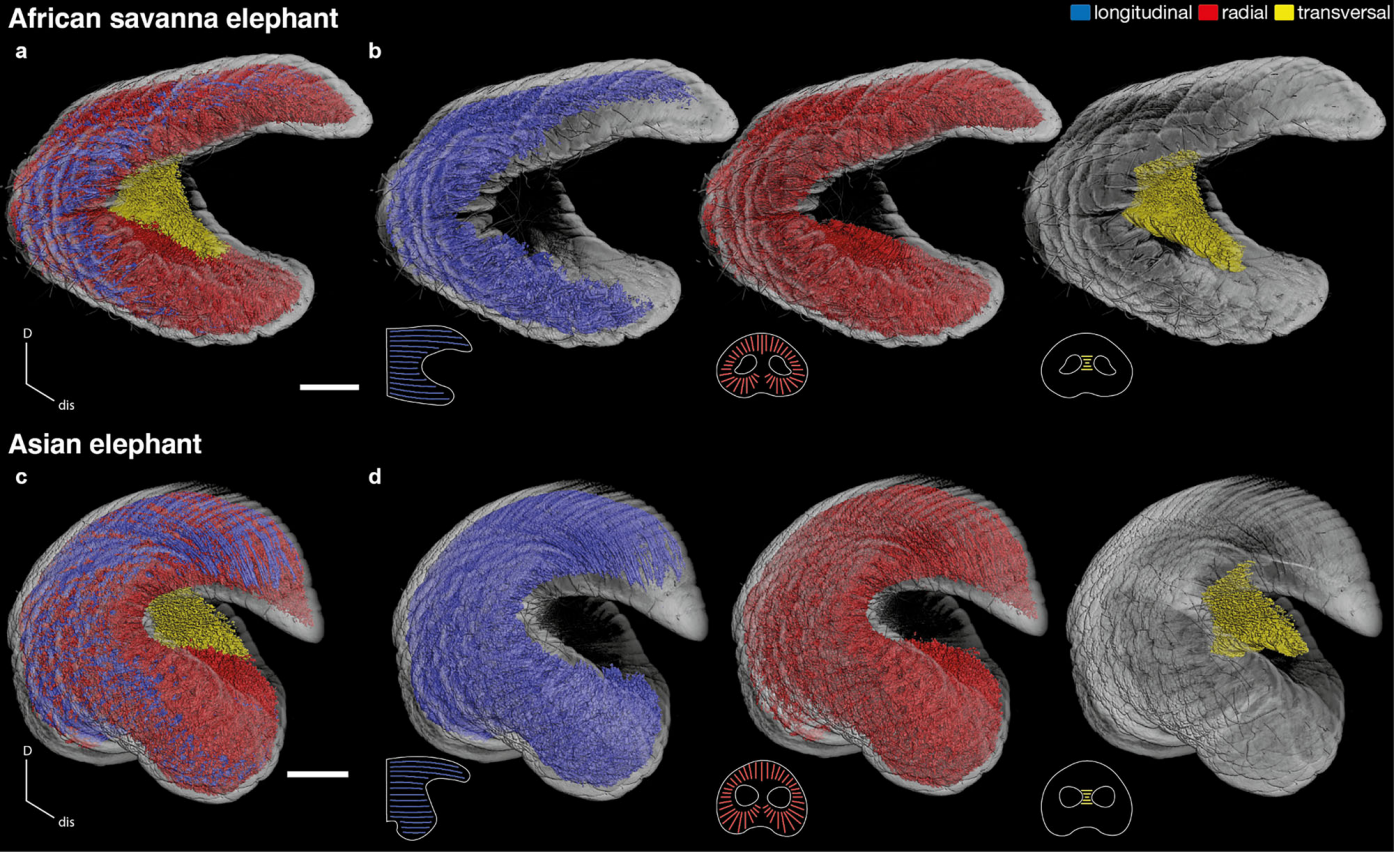
Longitudinal, radial, and transversal muscle fascicles in African savanna (*L. africana)* and Asian elephant (*E. maximus*) trunk tips. **a** Volume rendering of *L. africana* hemi-trunk tip with muscle fascicles colored according to muscle fascicle types. D, dorsal; dis, distal. Scale bar, 20 mm. **b** Volume rendering of classified muscle fascicles in *L. africana* hemi-trunk tip. Schematics show orientation of muscle fascicle types. Scale bar, 20 mm. **c** Volume rendering of *E. maximus* hemi-trunk tip with muscle fascicles colored according to muscle fascicle types. Conventions as in (**a**). **d** Volume rendering of classified muscle fascicles in *E. maximus* hemi-trunk tip. Conventions as in (**b**).

The volume rendering of muscle fascicles in the trunk tip of *L. africana* shows that the trunk is dominated by radial muscle fascicles (red; Fig. 2a). Longitudinal muscle fascicles (blue) in *L. africana* run parallel to the long axis of the trunk tip. Radial muscle fascicles (red) are orthogonal to the long axis of the trunk tip and are oriented around the nostrils, while transversal muscle fascicles are situated horizontally between the nostrils (Fig. 2b). The volume rendering of *E. maximus* trunk tip musculature shows that longitudinal muscle fascicles are very abundant (Fig. 2c). Like the *L. africana* trunk tip, longitudinal muscle fascicles (blue) run parallel to the long axis of the trunk tip (Fig. 2d). Radial muscle fascicles (red) are oriented orthogonally around and transversal muscle fascicles (yellow) between the nostrils (Fig. 2d).

### African savanna elephants have larger fraction of radial and transversal muscle fascicles than Asian elephants

**Fig. 3:**
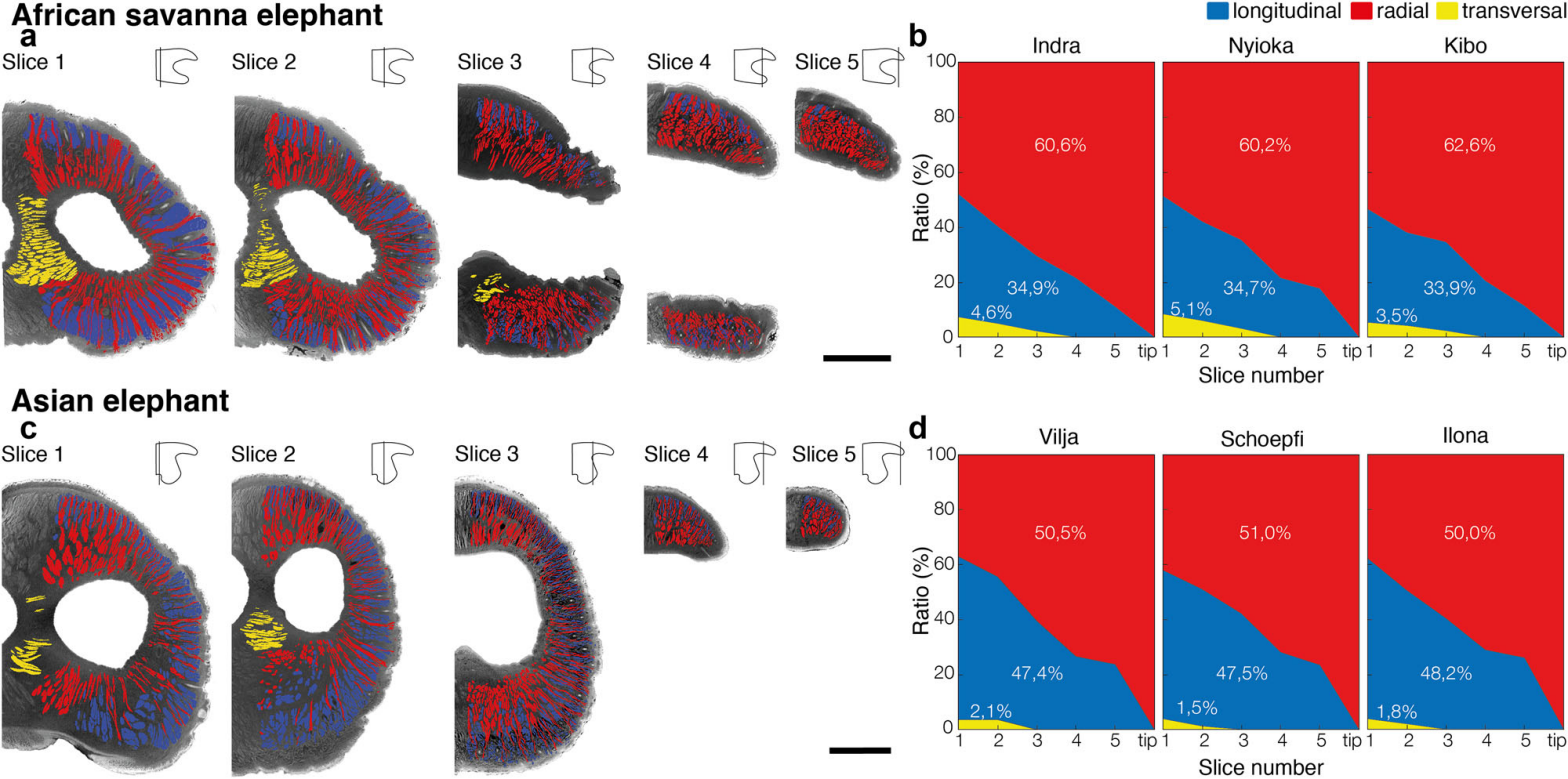
African savanna elephants (*L. africana*) have a larger fraction of radial muscle fascicles than Asian elephants (*E. maximus*). **a** Five transverse cross-sections of *L. africana* trunk tip with classified muscle fascicle segmentations overlayed. Schematics show where the respective cross-sections were taken. **b** Ratio of muscle types for *L. africana* trunk tip for five cross-sections. Total percentage of muscle types for all five slices in white letters. **c** Five transverse cross-sections of *E. maximus* trunk tip with classified muscle fascicle segmentations overlayed. Schematic shows where the respective cross-section was taken. **d** Ratio of muscle types for *E. maximus* trunk tips for five cross-sections. Total percentage of muscle types for all five slices in white letters. Scale bars (**a**, **c**), 20 mm.

*L. africana* and *E. maximus* differ in the relative fraction of muscle fascicle types (Fig. 3). The most proximal part of the *L. africana* trunk tip has a ratio of 1 to 1 between longitudinal and radial muscle fascicles (Fig. 3a). All three specimens of *L. africana* show similar ratios of longitudinal, radial, and transversal muscle fascicles for all five slices analyzed (Fig. 3b). In the most proximal part of the *L. africana* trunk tip longitudinal and radial muscle fascicles have the same ratio (~45%; Fig. 3b) and get progressively more radial towards the very trunk tip (~90% radial). Compared to *L. africana*, the *E. maximus* trunk tip has a larger fraction of longitudinal than radial muscle fascicles in the proximal part and contains a larger fraction of radial muscle fascicles towards the trunk tip (Fig. 3c). Different slices of the trunk tip in three *E. maximus* specimens show that the proximal trunk tip contains more longitudinal (60%) than radial (35%) muscle fascicles and is comparatively more longitudinal in the most distal part of the trunk tip compared to the three trunk tips of *L. africana* (25% longitudinal; Fig. 3d). The ratio of transversal muscle fascicles in the trunk tips of *L. africana* and *E. maximus* differs fivefold (Fig. 3d). Transversal muscle fascicle ratio is highest in the most proximal slice and gets lower towards the tip of the trunk. While in *L. africana* transversal muscle fascicles are still present in the third slice, in the *E. maximus* trunk tips transversal muscle fascicles go only as far as the second slice (Fig. 3a, c). In all three specimens of *L. africana*, the ratio of transversal muscle fascicles is much higher (average of 4.4%) than in the three *E. maximus* specimens (average of 1.8%; Fig. 3b, d). Overall, longitudinal muscle fascicles make up ~33% in *L. africana* and ~50% in *E. maximus* trunk tips, while radial and transversal muscle fascicles make up ~66% in *L. africana* and ~50% in *E. maximus* trunk tips.

### Longitudinal muscle fascicles differ between African and Asian elephant trunk tips

**Fig. 4:**
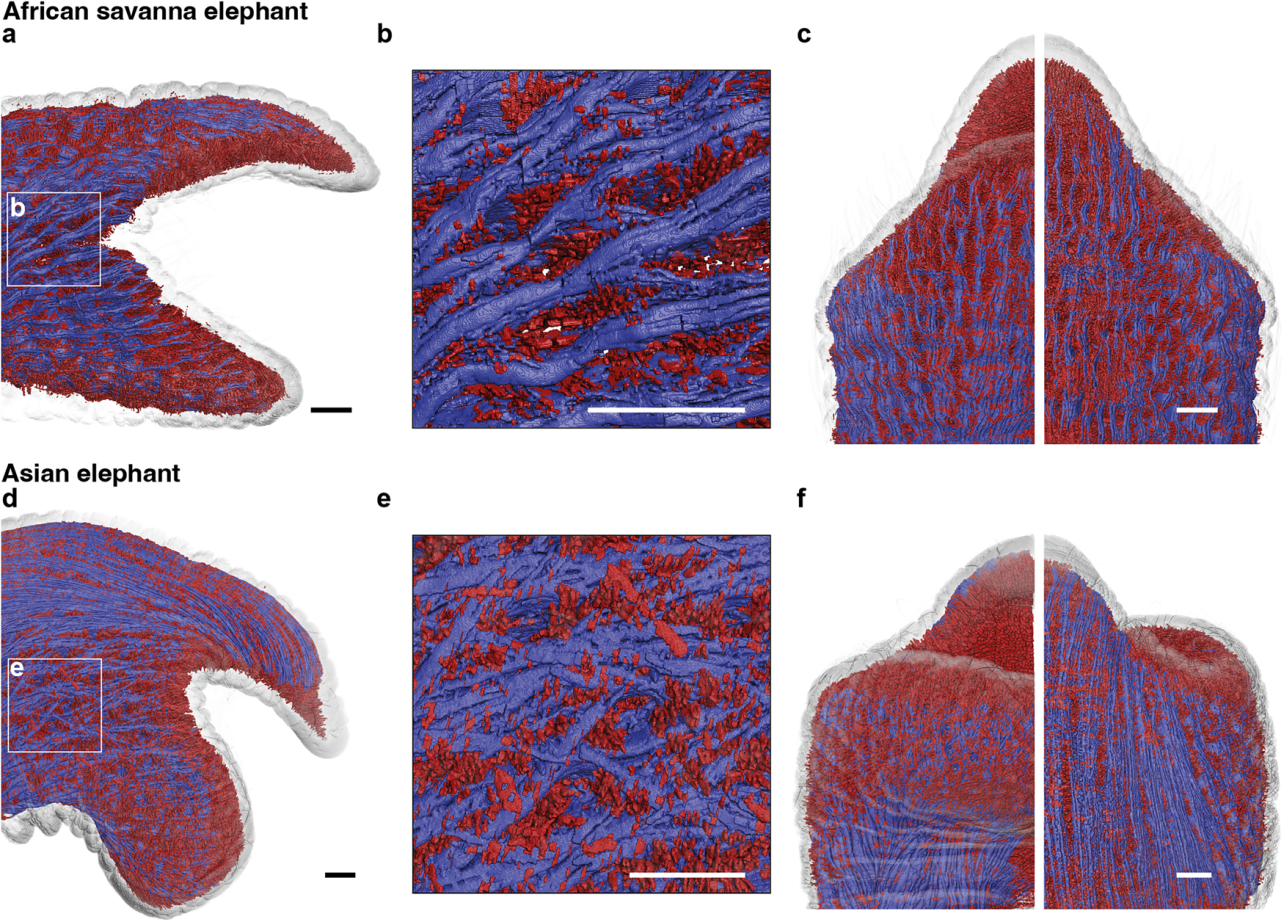
Longitudinal muscle fascicles differ in architecture between African savanna (*L. africana)* and Asian elephant (*E. maximus*) trunk tips. **a** Lateral view of volume rendering of longitudinal and radial muscle fascicles in *L. africana* trunk tip (Indra). **b** Close-up of cross-hatched longitudinal muscle fascicles on lateral side of *L. africana* trunk tip. **c** Ventral (left) and dorsal (right) view of volume rendering of longitudinal and radial muscle fascicles in *L. africana* trunk tip (Indra). **d** Lateral view of volume rendering of longitudinal and radial muscle fascicles in *E. maximus* trunk tip (Schoepfi). **e** Close-up of cross-hatched longitudinal muscle fascicles on lateral side of *E. maximus trunk tip*. **f** Ventral (left) and dorsal (right) view of volume rendering of longitudinal and radial muscle fascicles in *E. maximus* trunk tip (Schoepfi). Scale bars in all panels, 10 mm.

Longitudinal muscle fascicles in *L. africana* are on the outer layer of the trunk (Fig. 4a) and laterally intersect, building an interconnected net in the corner of the trunk opening (Fig. 4b). Specifically, in the corner of the trunk tip opening we observe longitudinal muscle fascicles that run towards the dorsal and ventral fingertip in *L. africana* (Fig. 4a, b) and the dorsal fingertip and the ventral bulb in *E. maximus* (Fig. 4d, e). This organization deviates from the otherwise parallel organization of muscle fascicles like the longitudinal muscle fascicles in the elephant trunk (Fig. 4c; Reference 14). Longitudinal muscle fascicles in the *E. maximus* trunk tip are on the outer layer of the trunk as well (Fig. 4d) and muscle fascicle strands also build an interconnected net (Fig. 4e). It appears that the crisscross arrangement of longitudinal muscle fascicles in the corner of the trunk tip opening enables these muscles to pull on the trunk fingers. On the ventral side, longitudinal muscle fascicles form a strong ventral bulb by overlapping from lateral to medial (Fig. 4f; left). In the dorsal finger, similar to the *L. africana* trunk tip, longitudinal muscle fascicles run parallel to the long axis of the trunk (Fig. 4f; right).

### Backward clamping with the ventral bulb and its muscular substrate in Asian elephants

**Fig. 5:**
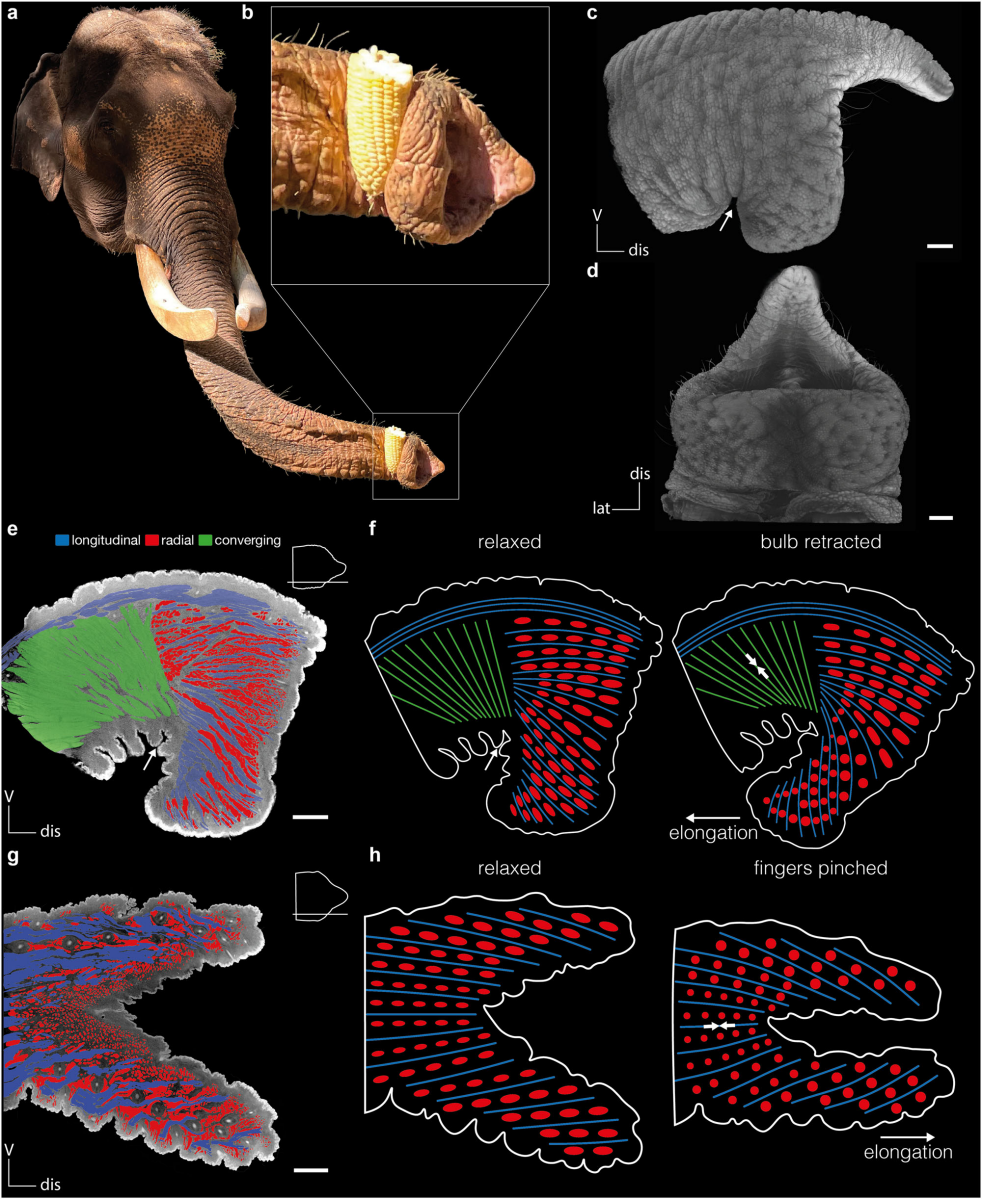
Backward clamping with ventral bulb and its muscular substrate in Asian elephants (*E. maximus*). **a** *E. maximus* clamps corn cob between trunk and ventral bulb. Credit: Lena Kaufmann. **b** Close-up of *E. maximus* trunk tip. The corn cob is clamped between the ventral side of the trunk and the ventral bulb of the trunk tip. **c** Lateral view of volume rendering of *E. maximus* trunk tip. Arrow indicates grove of ventral bulb. **d** Ventral view of volume rendering of *E. maximus* trunk tip. **e** Lateral cross-section of *E. maximus* trunk tip. Longitudinal muscle fascicles on dorsal side (blue), converging longitudinal muscle fascicles (green), and radial muscle fascicles (red). Schematic shows where the respective cross-section was taken. Arrow indicates grove of ventral bulb. **f** Left, schematic of lateral cross-section of *E. maximus* trunk tip in relaxed state. Right, retracted state. When converging longitudinal (green) and radial (red) muscle fascicles in the ventral bulb contract, the ventral bulb elongates and retracts to the proximal direction of the trunk. **g** Lateral cross-section of *L. africana* trunk tip. Longitudinal muscle fascicles (blue) run parallel to the long-axis of the trunk tip. Radial muscle fascicles (red) intersect the longitudinal muscle fascicles. **h** Left, schematic of lateral cross-section of *L. africana* trunk tip in relaxed state. Right, Pinched state. When longitudinal (blue) and radial (red) muscle fascicles (red) in the ventral bulb contract, the ventral bulb elongates and retracts to the proximal direction of the trunk. V, ventral; dis, distal; lat, lateral. Scale bars (**c**, **d**, **e**, **g**), 10 mm.

*E. maximus* has a ventral bulb that is used for clamping objects by putting the distal part of the trunk around the object, tightening the grip and folding down and extending the ventral bulb (Fig. 5a, b). We have often observed such a behavior in *E. maximus*, but we have never seen this in *L. africana*. We therefore wonder if the backwards flipping of the ventral bulb is a behavior specific to *E. maximus*.

While the dorsal fingers in *L. africana* and *E. maximus* trunk tips look quite similar, the ventral bulb in *E. maximus* looks like a shortened and thickened ventral finger in a *L. africana* trunk tip (Fig. 5c, d). When looking at cross sections of the *E. maximus* trunk tip, we see specialized longitudinal muscle fascicles (green) that ‘break’ at the ventral bulb and go around it, while radial muscle fascicles run orthogonally to it (Fig. 5e). They run from the dorsal to the ventral side of the trunk and in contrast to other trunk muscle fascicles, which have a parallel (longitudinal) or almost parallel arrangement, these muscle fascicles converge to the deep ridge, dividing the ventral bulb from the rest of the trunk. When these fascicles contract, the ventral bulb retracts (Fig. 5f). Together with the radial muscle fascicles, in the most distal part of the trunk tip, which elongate the bulb when contracting, it allows the elephant to clamp objects in between the ventral side of the trunk and the ventral bulb (Fig. 5f). These specialized lateral converging muscle fascicles are exclusively present in *E. maximus* (Fig. 5e), but not in *L. africana* (Fig. 5g). In the trunk tip of *L. africana*, longitudinal muscle fascicles (blue) pull at the point between the fingers and radial muscle fascicles (red) elongate them, allowing the elephant to pinch (Fig. 5h).

## Discussion

We traced and classified muscle fascicles in trunk tips of three *L. africana* and three *E. maximus* specimens by a combination of manual and automatic segmentation. Muscles were similar within elephant species but differed between species. *E. maximus* has a noticeably larger fraction of longitudinal muscles than *L. africana*, which might aid the trunk wrapping behavior that is more prevalent in *E. maximus*^5^. *L. africana* has two prehensile fingers, dorsal and ventral, which are similar in their muscular structure. In contrast, the *E. maximus* trunk tip has one dorsal finger and a ventral bulb. We describe backwards clamping behavior of the ventral trunk bulb in *E. maximus* and its putative muscular substrate.

Basic aspects of muscular organization were similar in *L. africana* and *E. maximus*, which confirms earlier work^14,17,18^. Like primates preshape their hands during reach-to-grasp movements, elephants have been observed to preshape their trunk tips according to object type^19^. The large number of independently controllable muscle fascicles in the elephant trunk tip is one of the most obvious differences to the primate hand, which contains only a small number of independent muscles^20–22^ controlled by more than a thousand motor neurons^23^. We observe also a predominance of small radial muscle fascicles in the trunk fingertip, a finding that replicates our earlier observations in an *E. maximus* calf trunk finger^14^. This suggests that muscle miniaturization and radial muscles play a key role for dexterous finger movements.

Another unique feature that we observed in the trunk tips of both elephant species is the intertwining of lateral longitudinal muscle fascicles. Typically, muscle fascicles in the elephant trunk and other muscular hydrostats, such as octopus and squid arms, are organized in parallel strands^7,8^. Here, however, longitudinal muscle fascicles on the lateral side cross each other to build an interconnected net, likely to pull on the trunk fingers. We reckon that these oblique muscle strands selectively pull on the trunk fingers and contribute to their immense mobility. This is likely advantageous for picking up small food items from the floor such as fruits, leaves and roots, which are available in the environment of both species. The force that the trunk tip fingers exert is relatively small, further suggesting that this part of the trunk is dedicated to precise grasping of objects^24^.

Our data provide strong indications for species differences in trunk tip muscle architecture between *L. africana* and *E. maximus*. Specifically, muscle fascicle types were very similar within species, but differed markedly between species. This conclusion has to be taken with a grain of salt given the small size of our sample (three *L. africana* and three *E. maximus* specimens). Given the small sample size, we also refrained from a formal statistical evaluation. We also did not take the absence or presence, nor the morphology of tusks into account, which have been shown to influence the grasping technique^25^. However, we do not believe that this influenced the general trunk morphology.

The most noticeable difference between *L. africana* and *E. maximus* trunk tip musculature was the larger fraction of longitudinal muscle fascicles in *E. maximus*. We think that the predominance of longitudinal muscle fascicles in *E. maximus* compared to *L. africana* is likely related to the tendency of *E. maximus* to wrap their trunk around rather than to pinch objects^6^. Bending of the trunk is achieved through the interplay of longitudinal, radial, and transversal muscle fascicles. First, the central core of transversal and radial muscle fascicles causes the trunk to protrude, followed by the contraction of dorsal longitudinal muscle fascicles while transversal and radial muscle fascicles are still active, which leads to upward bending of the trunk^8^. If the lateral longitudinal muscle fascicles would contract instead of the dorsal longitudinal muscle fascicles, the trunk would bend sideways. Without the activity of radial and transversal muscle fascicles the trunk would only retract. Due to their arrangement around the outer layer of the trunk, longitudinal muscle fascicles develop more bending moment than the inner layer of radial muscle fascicles^8^. This could be a reason why we see – especially dorsally and ventrally –a higher ratio of longitudinal muscle fascicles in *E. maximus*. These very prominent ventral longitudinal muscle fascicles most likely allow *E. maximus* to roll in the trunk (by shortening the ventral part of the trunk) in the context of object wrapping behavior. *L. africana*, however, primarily use a pinching behavior which necessitates a higher extension of the trunk. Hence, they have a higher abundance of radial and transversal muscle fascicles which make up two thirds of their trunk tip vs. half of the trunk tip in *E. maximus*.

Differences in trunk tip morphology may have resulted from different feeding behaviors of *E. maximus* and *L. africana* due to the different ecosystems they inhabit. *L. africana* mainly live in grasslands, savannas and deserts, while *E. maximus* live in moist or dry tropical forests^26^. Trunks of *L. africana* are tougher with much thicker whiskers, fewer and more marked wrinkles and they operate in a very tip-centered pinching mode. This might be advantageous for more precise pickings in the open savanna and desert landscapes they live in. The *E. maximus* trunk is overall more flexible and its wrapping and ventral bulb backwards flipping abilities include more of the trunk in food acquisition. This behavior might be advantageous for picking up bulk foliage in the dense vegetation of tropical rain forests they live in. However, how such different motor abilities relate to feeding is less clear and warrants more behavioral data.

A behavior that we observed exclusively in *E. maximus* is the backward clamping, where the elephants clamp objects between the ventral side of their trunk and their ventral bulb. First, the elephant wraps around the object with its trunk tip, then extends its ventral bulb and finally clamps the object between its extended ventral bulb and the ventral side of the trunk. This behavior most likely is achieved by specialized converging longitudinal muscle fascicles that run from the dorsal side of the trunk tip to the grove of the ventral bulb. We hypothesize that the backwards clamping of the ventral trunk tip bulb is achieved by simultaneously contracting the lateral converging muscle fascicles and elongating the ventral bulb, which is driven by contracting the radial muscle fascicles in the trunk tip bulb. *E. maximus* is known to browse, ripping leaves and twigs from trees and shrubs, while *L. africana* predominantly grazes grass and ground-level plants. This suggests that the backward flipping behavior of *E. maximus* is a helpful adaptation for ripping leaves from trees and the very dexterous and fine-tuned fingertips of *L. africana* help to pick up leaves and small plants from the ground.

The ~63,000 facial nucleus neurons in *L. africana*, compared to ~54,000 in *E. maximus*, outnumber all other land-living animals^27^. The trunk tip fingers in *L. africana* are very prominently represented as motor fovea in the highly differentiated facial nucleus^27^. These regions of increased cell density have a striking resemblance with the trunk tip fingers. Kaufmann et al. (2022) suggest that having a trunk tip motor fovea aligns with the behavior of *L. africana* which use their fingers to constantly probe their environment^6^, with the highly tactile trunk innervation^3^, as well as with specializations of the trunk tip^4^. However, facial motor neuron number is only one predictor of facial motor ability^5^.

Due to the almost infinite degrees of freedom in muscular hydrostats, it is crucial to integrate external and internal sensory information for assessing which motor action is necessary. In the octopus arm, another highly tactile muscular hydrostat, ~3 Mio motor neurons^28^ innervate the densely packed muscle fascicles. Axons associated with transmitting motor commands innervate a large pool of motor neurons *en passant*, which implies that central and peripheral motor commands involve the simultaneous recruitment of large groups of motor neurons along the arm^29^. Furthermore, a unique interplay between global central commands and local sensory signals may determine the site of movement initiation^29^. Whether elephant trunk motor control works in a similar fashion is not clear yet. A detailed analysis of musculature and its neural representation will give more insight into motor control of the trunk.

We segmented *L. africana* and *E. maximus* trunk tip musculature. Our detailed reconstructions reveal that both species operate the trunk tip with a large number of predominantly small muscle fascicles. Trunk tips of *L. africana* have a ratio of ~2:1 of radial to longitudinal muscle fascicles, while *E. maximus* have a ratio of ~1:1 of radial to longitudinal muscle fascicles. Species differences in trunk tip muscle architecture reflect preferences of *L. africana* for object pinching and *E. maximus* for object wrapping, respectively. Furthermore, trunk tips of *E. maximus* have specialized converging longitudinal muscle fascicles that run from the dorsal side of the trunk tip to the grove of the ventral bulb. These specialized muscle fascicles most likely allow them to clamp objects between their ventral bulb and the ventral side of their trunk, a behavior that is likely the equivalent to pinching in *L. africana*.

## Materials and methods

### Elephant specimens

#### Sample collection

The elephant specimens used for our analysis came from zoo elephants collected by the IZW (Leibniz Institute for Zoo and Wildlife Research Berlin, Germany) in strict accordance with CITES (Convention on International Trade in Endangered Species of Wild Fauna and Flora). The animals included in the study were euthanized by experienced zoo veterinarians due to insurmountable health complications. According to the veterinarians that euthanized the elephants, the trunk tips did not appear to be affected by the health situation of the individuals. Please see Table 1 for more information on elephant specimens used in this study.

**Table 1:** Overview of elephant specimens used for this study

Name (Species)	Sex	Age (years), died on (dd/mm/yyyy)	Location at birth	Location at death	Specimen treatment
Indra (*L. africana*)	Female	34, 06/09/2022	Unknown, Africa - Wild	Elefantenhof Platschow, Germany	Formalin
N’Yoka (*L. africana*)	Female	44, 07/07/2022	South Africa, Africa - Wild	Knutheborg Safaripark, Denmark	–20 °C
Kibo (*L. africana*)	Male	45, 08/07/2022	Zoo Hannover, Germany	Bioparc Valencia, Spain	–20 °C
Vilja (*E. maximus*)	Female	60, 10/07/2010	Indonesia, Asia - Wild	Zoo Stuttgart, Germany	–20 °C
Schöpfi (*E. maximus*)	Female	50, 03/02/2010	India, Asia - Wild	Zoo Dresden, Germany	–20 °C
Ilona (*E. maximus*)	Female	45, 31/03/2014	Unknown, Asia - Wild	Zoo Karlsruhe, Germany	–20 °C

African savanna elephant*, Loxodonta africana*. Data obtained from two female and one male *L. africana* specimens was used for this study.

#### Indra

(34 y) This *L. africana* cow was born between the years of 1986-1988 (the exact date is unknown) in the wild. She has spent her life as a performer at the shows of Circus Zavatta and was moved to Elefantenhof Platschow, Germany. Indra died of a Salmonella infection in June 2022, that led to a ruptured intestine.

#### N’Yoka

(44 y) This *L. africana* cow was born 1978 in Krüger-Nationalpark, South Africa. She was moved to Borås Djurpark, Sweden on May 20, 1979. On June 22, 2021 N’Yoka was moved again to Knutheborg Safaripark, Denmark where she was euthanized due to an illness on July 07, 2022.

#### Kibo

(45) This *L. africana* bull was born November 27, 1977 in the Zoo Hannover, Germany. He was moved to Borås Djurpark, Sweden on May 5, 1986 and then to Bioparc Valencia, Spain on September 19, 2013. Kibo died July 8, 2022.

Asian elephant*, Elephas maximus*. Data obtained from three female *E. maximus* specimens was used for this study.

#### Vilja

(60 y) This *E. maximus* cow was born ca. 1949 in Sumatra and came to the Zoo Stuttgart, Germany in 1952. She died as a result of a circulatory collapse on July 10, 2010.

#### Schöpfi

(50 y) This *E. maximus* cow was born ca. 1959 in Assam, India and came to the Zoo Dresden, Germany on October 7, 1960 where she lived with a group of *L. africana*, which could have altered the grasping strategy due to social imitation. However, we do not think this affected the basic muscle anatomy of this specimen, as the muscle anatomy in this individual looked similar to that of the other specimens of this species. Because of an inflammation in her foot she was not able to stand and was euthanized on February 3, 2010.

#### Ilona

(45 y) This *E. maximus* cow was born ca. 1969 and lived in a circus until 1983. She was moved to the Zoo Hannover, Germany on July 2, 1983, then to the Zoo Heidelberg, Germany on September 28, 2004, and finally to the Zoo Karlsruhe, Germany on November 19, 2009. Because of persistent pain due to arthrosis in her front ankles and her right knee she was euthanized on March 31, 2014.

### Elephant trunk tip preparation and staining

#### Sample condition

After death of the elephants, trunk tips were either frozen at -20 °C or fixed in 4% formaldehyde. The trunk tips did not seem to be damaged or affected by the cause of death.

#### Sample preparation

We used six trunk tips (three adult *L. africana* specimens, three adult *E. maximus* specimens) for this study. The trunk tips were allowed to thaw for a day and then cut parasagitally. To facilitate the staining procedure we decided to focus on hemi-trunk tips for analysis. We arbitrarily chose the left hemi-trunk tip to allow for a better comparison between specimens. For specimens with injuries on the left hemi-trunk tip we used the right hemi-trunk tip.

#### Sample staining

For contrast enhancement of the tissue, the hemi-trunk tips were iodine-stained. Staining was done by submersing specimens in 1% Lugol’s iodine solution in water for 6 – 8 weeks, dependent on the size of the trunk tips^30^.

### Trunk tip microCT scanning and preprocessing

Trunk tips were scanned with a YXLON FF20 system (YXLON International GmbH, Hamburg, Germany) at Humboldt University of Berlin, Germany. The scanner is equipped with a Perkin Elmer Y Panel 4343 CT detector and a nano-focus x-ray transmission tube operating at 190 kV.

### Manual segmentation of trunk tip muscle fascicles

Labeling of trunk tip muscle fascicles was performed in the Amira Software (AmiraZIBEdition 2023, 2024, 2025 Zuse Institute Berlin). For training of the automated segmentation algorithm, we segmented patches of muscle fascicles in ten slices per axis per elephant trunk tip using a combination of the ‘Lasso’ and ‘Brush’ modules to manually annotate muscle fascicles. We then manually classified these individual fascicles for training of the automated classification algorithm.

### Automated binary segmentation using machine learning algorithm

For binary segmentation we used a UNet + +^31^ architecture, an extension of the popular U-Net^32^, with an EfficientNet architecture^33^ as backbone. The model takes 2D images of the scan as input and predicts the corresponding binary segmentation mask. Every pixel is assigned either the label ‘muscle’ or ‘background’. The training was implemented using PyTorch^34^ and PyTorch Lightning^35^, models by Segmentation Models Pytorch^36^, and image augmentation by Albumentations^37^.

A partial manual segmentation of the trunk tip served as the training data. To prepare the data for training, we selected such areas that are already completely and correctly segmented. The final training data was around 30 regions for each trunk across multiple slices and planes. Next, we clipped the values of the voxels in the same range as a human annotator would for every scan. Finally, the data was tiled into 128 × 128-pixel sized squares. To increase the small amount of available training data, we applied various transformations to augment the data, including reflecting (horizontally or vertically), rotating, adding noise and blurring the image. Every transformation was applied to a tile after sampling during training with a probability of 0.5. Exact hyperparameters are given in Tables 2, 3. The final model is trained on the combined data of all elephants.

**Table 2:** Hyperparameters used for automated segmentation

Hyperparameter	Value
Training Loss	Binary Cross-Entropy
Epochs	77 (early stopping)
Batch size	32
Backbone	EfficientNet-B4
Optimizer	Adam
Learning Rate	0.001
Learning Rate Schedule	Reduce On Plateau
Factor	0.1
Patience	5
Padding of regions (before tiling)	42 pixels
Padding mode	Reflect
Tiling	
Overlap of tiles	42 pixels

**Table 3:** Parameters of data augmentation transformations

Blur	
Max. kernel size	5
Gaussian Noise	
Mean	0
Variance	10
Rotation	
Max. angle	45 degrees
Fill edges	Reflect 101

After training, the model was applied to the complete data set. As the model only takes 2D data as input, all slices along a single axis were tiled, and the patches were passed into the model. In some instances, to improve the segmentation, we repeated this procedure for the other axes. In this case, to combine the resulting three predictions, the logical OR operation is applied per pixel. Meaning, if the pixel is classified as a muscle in at least one of the planes, it is labeled as a muscle in the final prediction.

Manual and automated segmentation results appear similar, and are also consistent with previous anatomical studies^17,18^. Segmentation results were remarkably consistent within each species. However, we observed strong differences between the two species (see Fig. 2). These results suggest that our segmentation reveals interspecific variation in trunk muscular anatomy.

### Automated classification using machine learning algorithm

For the purpose of muscle classification, the popular machine-learning model nnU-Net^38^ has been used. nnU-Net is a semantic segmentation tool that automatically adapts to a given dataset. It is a supervised learning model, which means that the training examples need to be provided by the user. It works with 2D and 3D data and can accept multiple channels/modalities of the image.

Classification of muscle fascicles was done in multiple steps: (1) downsampling of the image, (2) normalization, (3) training, (4) prediction, and (5) upsampling.

#### Downsampling

The image size varies across the different samples. To ensure uniform image size in the XY dimensions, enhance performance, and mitigate memory issues during the training of the segmentation model and muscle type prediction, the resolution of the images was downsampled in the XY plane to a resolution of [350 350 Z] voxels using *Amira*. The same resolution was used for model training and prediction.

#### Normalization

Both training data and data for prediction are normalized using local normalization^39^, implemented in *Amira*. Local normalization ensures uniform mean and variance in the image’s local neighborhood. This type of normalization is particularly helpful for correction of uneven intensities and shading artefacts. This is particularly beneficial given a non-uniform staining of some trunk tips, which can lead to poor contrast in areas with less staining.

#### Training

Model training (and later prediction), was performed in the 3D configuration. Training data consists of manually annotated muscle fascicles of an *E. maximus* calf^14^ and the *E. maximus* specimen *Schöpfi*. In the case of the first, 380 slices were manually annotated, while the later consisted of 140 slices (in different areas of the trunk). There are three different types of muscle fascicles in the trunk: radial, longitudinal and transversal. Each muscle fascicle type had a different label assigned to it.

nnU-Net automatically extracts “dataset fingerprint” and determines “rule-based” parameters, such as patch size, batch size, network topology, and the number of pooling operations. Other parameters are “fixed” and independent of the input images. These include the optimizer, loss function, data augmentation procedure, learning rate, and other training hyperparameters as defined in the original nnU-Net framework. Both, rule-based and fixed parameters can be adjusted manually. The nnU-Net preprocessing script estimated a patch size of [320 320 20] voxels, which was manually adjusted to [350 350 20] voxels to cover the entire size of the sample in the XY plane, as well as contain multiple slices in the Z direction. A large patch size in XY allows the network to learn spatial relations between different muscle types, while containing multiple slices in Z helps the network to learn about the morphology of the different muscle fascicle types. Before running the training or prediction, the images were standardized using z-score. Other training parameters remained unchanged from the automatic estimation.

#### Prediction

Multiclass segmentation is performed on the input image. Each voxel is classified as either background or one of the three muscle fascicle types (radial, longitudinal, transversal) based on the model’s predictions.

Despite training being performed solely on specimens of *E. maximus* and an *E. maximus calf*, the model could also be used for the *L. africana* specimens, thanks to similarities in trunk anatomy^5^.

#### Upsampling

After prediction, upsampling back to the original size was performed within *Amira*. After upsampling, any potential wrongly assigned areas had to be manually corrected for analysis.

### Statistics and reproducibility

Data was analyzed using Excel (Microsoft). The calculations of elephant trunk cross-sectional areas in Fig. 3 as well as visualizations in all figures were done in AmiraZIBEdition (Version 2025). Data was plotted with MATLAB (Version R2024b). Figure layouts were prepared with Adobe Illustrator (Version 29.8).

### Reporting summary

Further information on research design is available in the Nature Portfolio Reporting Summary linked to this article.

## Supplementary information

Reporting Summary

## Data Availability

The source data used to plot Fig. 3b, 3d can be found data on https://gin.g-node.org/elephant/trunk_tip. This paper does not report original code. Any additional information required to reanalyze the results of the paper is available from the lead contact (michael.brecht@bccn-berlin.de) upon reasonable request.

## References

[1] Schulz, A. K. et al. Elephant trunks use an adaptable prehensile grip. *Bioinspir. Biomim.* **18**, 026008 (2023). 10.1088/1748-3190/acb477 36652720

[2] Schulz, A. K. et al. Suction feeding by elephants. *J. R. Soc. Interface* **18**, 20210215 (2021). 34062103 10.1098/rsif.2021.0215 PMC8169210

[3] Purkart, L. et al. Trigeminal Ganglion and Sensory Nerves Suggest Tactile Specialization of Elephants. *Curr. Biol.* **32**, 904–910.e3 (2022). 35063122 10.1016/j.cub.2021.12.051

[4] Rasmussen, L. E. L. & Munger, B. L. The sensorineural specializations of the trunk tip (finger) of the Asian elephant, *Elephas maximus*. *Anat. Rec.* **246**, 127–134 (1996). 8876831 10.1002/(SICI)1097-0185(199609)246:1<127::AID-AR14>3.0.CO;2-R

[5] Nabavizadeh, A. Of tusks and trunks: A review of craniofacial evolutionary anatomy in elephants and extinct. *Proboscidea. Anat. Rec.* (2024). 10.1002/ar.25578 39380178

[6] Racine, R. N. Behavior associated with feeding in Captive African and Asian Elephants. *Elephant* **1**, 57–71 (1980).

[7] Kier, W. M. & Smith, K. K. Tongues, Tentacles and Trunks: The Biomechanics of Movement in Muscular-Hydrostats. *Zool. J. Linn. Soc.* **83**, 307–324 (1985).

[8] Smith, K. K. & Kier, W. M. Trunks, tongues, and tentacles: Moving with skeletons of muscle. *Am. Sci.* **77**, 28–35 (1989).

[9] Kier, W. M. & Stella, M. P. The arrangement and function of octopus arm musculature and connective tissue. *J. Morphol.* **268**, 831–843 (2007). 17624930 10.1002/jmor.10548

[10] Kier, W. M. The diversity of hydrostatic skeletons. *J. Exp. Biol.* **215**, 1247–1257 (2012). 22442361 10.1242/jeb.056549

[11] Di Clemente, A., Maiole, F., Bornia, I. & Zullo, L. Beyond muscles: role of intramuscular connective tissue elasticity and passive stiffness in octopus arm muscle function. *J. Exp. Biol.* **224**, jeb242644 (2021). 34755832 10.1242/jeb.242644

[12] Zullo, L., Di Clemente, A. & Maiole, F. How octopus arm muscle contractile properties and anatomical organization contribute to arm functional specialization. *J. Exp. Biol.* **225**, jeb243163 (2022). 35244172 10.1242/jeb.243163

[13] Gilbert, R. J., Napadow, V. J., Gaige, T. A. & Wedeen, V. J. Anatomical basis of lingual hydrostatic deformation. *J. Exp. Biol.* **210**, 4069–4082 (2007). 18025008 10.1242/jeb.007096

[14] Longren, L. L. et al. Dense reconstruction of elephant trunk musculature. *Curr. Biol.* **33**, 4713–4720 (2023). 37757829 10.1016/j.cub.2023.09.007

[15] Dagenais, P., Hensman, S., Haechler, V. & Milinkovitch, M. C. Elephants evolved strategies reducing the biomechanical complexity of their trunk. *Curr. Biol.* **31**, 4727–4737 (2021). 34428468 10.1016/j.cub.2021.08.029

[16] Olson, W., Zhang, L., O’Connor, D. H. & Kleinfeld, D. Elephant trunks: Strength and dexterity from mini-fascicles. *Curr. Biol.* **33**, R1203–R1205 (2023). 37989101 10.1016/j.cub.2023.10.012 PMC11039408

[17] Cuvier, G. & MacGillivray, W. The animal kingdom of the baron Cuvier. *J Nat Hist*. **31** (1839).

[18] Shoshani, J. It’s a nose! It’s a hand! It’s an elephant’s trunk!. *Nat. Hist.* **106**, 36–45 (1997).

[19] Soppelsa, J. et al. The relationship between distal trunk morphology and object grasping in the African savannah elephant (*Loxodonta africana*). *PeerJ* **10**, e13108 (2022). 35368332 10.7717/peerj.13108 PMC8969868

[20] Van Leeuwen, T., Vanhoof, M. J., Kerkhof, F. D., Stevens, J. M. & Vereecke, E. E. Insights into the musculature of the bonobo hand. *J. Anat.* **233**, 328–340 (2018). 29938781 10.1111/joa.12841 PMC6081514

[21] Vanhoof, M. J., Van Leeuwen, T. & Vereecke, E. E. The forearm and hand musculature of semi-terrestrial rhesus macaques (*Macaca mulatta*) and arboreal gibbons (Fam. Hylobatidae). Part I. Description and comparison of the muscle configuration. *J. Anat.* **237**, 774–790 (2020). 32511764 10.1111/joa.13222 PMC7495296

[22] Vanhoof, M. J., van Leeuwen, T., Galletta, L. & Vereecke, E. E. The forearm and hand musculature of semi-terrestrial rhesus macaques (*Macaca mulatta*) and arboreal gibbons (fam. Hylobatidae). Part II. Quantitative analysis. *J. Anat.* **238**, 321–337 (2021). 33011967 10.1111/joa.13314 PMC7812139

[23] Gesslbauer, B. et al. Axonal components of nerves innervating the human arm. *Ann. Neurol.* **82**, 396–408 (2017). 28833372 10.1002/ana.25018

[24] Costes, P. et al. Maximum trunk tip force assessment related to trunk position and prehensile’fingers’ implication in African savannah elephants. *Plos one* **19**, e0301529 (2024a). 38743734 10.1371/journal.pone.0301529 PMC11093316

[25] Costes, P. et al. Effect of the habitat and tusks on trunk grasping techniques in African savannah elephants. *Ecol. Evol.* **14**, e11317 (2024b). 38646004 10.1002/ece3.11317 PMC11027014

[26] Campos-Arceiz, A. & Blake, S. Megagardeners of the forest–the role of elephants in seed dispersal. *Acta Oecol* **37**, 542–553 (2011).

[27] Kaufmann, L. V., Schneeweiß, U., Maier, E., Hildebrandt, T. & Brecht, M. Elephant facial motor control. *Sci. Adv.* **8**, eabq2789 (2022). 36288305 10.1126/sciadv.abq2789 PMC9604532

[28] Young, J. Z. The anatomy of the nervous system of *Octopus vulgaris*. Oxford, UK: Clarendon Press (1971).

[29] Zullo, L., Eichenstein, H., Maiole, F. & Hochner, B. Motor control pathways in the nervous system of Octopus vulgaris arm. *J. Comp. Physiol.* **205**, 271–279 (2019). 30919046 10.1007/s00359-019-01332-6 PMC6478645

[30] Metscher, B. D. MicroCT for Comparative Morphology: Simple Staining Methods Allow High-Contrast 3D Imaging of Diverse Non-Mineralized Animal Tissues. *BMC Physiol.* **9**, 11 (2009). 19545439 10.1186/1472-6793-9-11 PMC2717911

[31] Zhou, Z., Siddiquee, M. M. R., Tajbakhsh, N. & Liang, J. Unet++: A nested u-net architecture for medical image segmentation,” Deep Learning in Medical Image Analysis and Multimodal Learning for Clinical Decision Support: 4th International Work-shop, DLMIA 2018, and 8th International Workshop, ML-CDS 2018, held in conjunction with MICCAI 2018, Granada, Spain, 11045, 3–11 (2018). 10.1007/978-3-030-00889-5_1 PMC7329239 32613207

[32] Ronneberger, O., Fischer, P. & Brox, T. U-net: Convolutional networks for biomedical image segmentation, *ArXiv*. abs/1505.04597 (2015).

[33] Tan, M. & Le, Q. V. Efficientnet: Rethinking model scaling for convolutional neural networks, *ArXiv*. abs/1905.11946 (2019).

[34] Paszke, A. et al. PyTorch: An Imperative Style, High-Performance Deep Learning Library. In *Advances in Neural Information Processing Systems* (eds Wallach, H. et al.) Vol. 32, 8024–8035 (2019).

[35] Falcon, W. PyTorch Lightning (2019).

[36] Iakubovskii, P. Segmentation models pytorch (2019).

[37] Buslaev, A. et al. Albumentations: Fast and flexible image augmentations. *Information* **11**, 125 (2020).

[38] Isensee, F., Jaeger, P. F., Kohl, S. A., Petersen, J. & Maier-Hein, K. H. nnU-Net: a self-configuring method for deep learning-based biomedical image segmentation. *Nat. Methods* **18**, 203–211 (2021). 33288961 10.1038/s41592-020-01008-z

[39] Sage, D. & Unser, M. Teaching image-processing programming in Java. *IEEE Signal Process. Mag.* **20**, 43–52 (2003).

