# Supplementary material for: Elephant trunk tip musculature reflects species differences in grasping behavior
Source: Commun Biol. 2025 Dec 8;8:1779. doi: 10.1038/s42003-025-08998-6 (PMC12715207; doi:10.1038/s42003-025-08998-6)
Supplement: Supplementary file 1 — Reporting Summary [file 42003_2025_8998_MOESM1_ESM.pdf]

nature portfolio

Corresponding author(s): Michael Brecht

Last updated by author(s): Sep 15, 2025

Reporting Summary

Nature Portfolio wishes to improve the reproducibility of the work that we publish. This form provides structure for consistency and transparency in reporting. For further information on Nature Portfolio policies, see our Editorial Policies and the Editorial Policy Checklist.

Statistics

For all statistical analyses, confirm that the following items are present in the figure legend, table legend, main text, or Methods section.

n/a	Confirmed	
☐	☒	The exact sample size (*n*) for each experimental group/condition, given as a discrete number and unit of measurement
☐	☒	A statement on whether measurements were taken from distinct samples or whether the same sample was measured repeatedly
☒	☐	The statistical test(s) used AND whether they are one- or two-sided<br>*Only common tests should be described solely by name; describe more complex techniques in the Methods section.*
☒	☐	A description of all covariates tested
☒	☐	A description of any assumptions or corrections, such as tests of normality and adjustment for multiple comparisons
☒	☐	A full description of the statistical parameters including central tendency (e.g. means) or other basic estimates (e.g. regression coefficient) AND variation (e.g. standard deviation) or associated estimates of uncertainty (e.g. confidence intervals)
☒	☐	For null hypothesis testing, the test statistic (e.g. *F*, *t*, *r*) with confidence intervals, effect sizes, degrees of freedom and *P* value noted<br>*Give P values as exact values whenever suitable.*
☒	☐	For Bayesian analysis, information on the choice of priors and Markov chain Monte Carlo settings
☒	☐	For hierarchical and complex designs, identification of the appropriate level for tests and full reporting of outcomes
☒	☐	Estimates of effect sizes (e.g. Cohen's *d*, Pearson's *r*), indicating how they were calculated

Our web collection on statistics for biologists contains articles on many of the points above.

Software and code

Policy information about availability of computer code

Data collection	Amira software (AmiraZIBEdition, 2023, 2024, and 2025 Zuse Institute Berlin)
Data analysis	Amira software (AmiraZIBEdition, 2023, 2024, and 2025 Zuse Institute Berlin)

For manuscripts utilizing custom algorithms or software that are central to the research but not yet described in published literature, software must be made available to editors and reviewers. We strongly encourage code deposition in a community repository (e.g. GitHub). See the Nature Portfolio guidelines for submitting code & software for further information.

Data

Policy information about availability of data

All manuscripts must include a data availability statement. This statement should provide the following information, where applicable:

- Accession codes, unique identifiers, or web links for publicly available datasets
- A description of any restrictions on data availability
- For clinical datasets or third party data, please ensure that the statement adheres to our policy

All data supporting the findings of this study are available within the paper and its Supplementary Information.

Research involving human participants, their data, or biological material

Policy information about studies with human participants or human data. See also policy information about sex, gender (identity/presentation), and sexual orientation and race, ethnicity and racism.

Reporting on sex and gender	n/a
Reporting on race, ethnicity, or other socially relevant groupings	n/a
Population characteristics	n/a
Recruitment	n/a
Ethics oversight	n/a

Note that full information on the approval of the study protocol must also be provided in the manuscript.

Field-specific reporting

Please select the one below that is the best fit for your research. If you are not sure, read the appropriate sections before making your selection.

☐ Life sciences ☐ Behavioural & social sciences ☒ Ecological, evolutionary & environmental sciences

For a reference copy of the document with all sections, see nature.com/documents/nr-reporting-summary-flat.pdf

Ecological, evolutionary & environmental sciences study design

All studies must disclose on these points even when the disclosure is negative.

Study description	We performed dense muscle fascicle reconstruction of three African and three Asian elephant hemi-trunk tips using a combination of manual and automated segmentation of high-resolution microfocus tomography (microCT) scans. We distinguish three types of muscle fascicles: longitudinal (bending and shortening of trunk), radial (elongating of trunk) and transversal muscle fascicles (elongating of nasal septum). In line with earlier work, we found that in both elephant species the trunk tip contains numerous tiny muscle fascicles. Furthermore, muscle fascicles in the distal trunk tip are predominantly radial. We found that Asian elephant trunk tips have markedly more longitudinal and fewer transversal muscle fascicles, as well as specialised muscle fascicles in the lateral trunk tip, which most likely reflects the stronger reliance on trunk bending and object wrapping in Asian elephant trunks.
Research sample	The elephant trunk specimens used for this analysis came from the IZW (Institute for Zoo and Wildlife Research). The authors were not involved in the euthanasia protocol. We included 3 specimens of Asian elephants (Elephas maximus) and three specimens of African savanna elephants (Loxodonta africana). The specimens were preserved in PFA and did not show obvious pathologies.
Sampling strategy	The specimens were sampled according to availability.
Data collection	Samples were scanned at the Humboldt-Universität zu Berlin with a YXLON FF20 CT scanner (YXLON, Hamburg, Germany). Scans for all samples were performed with helix scan sample rotation.
Timing and spatial scale	n/a
Data exclusions	No data were excluded.
Reproducibility	n/a
Randomization	n/a
Blinding	n/a

Did the study involve field work? ☐ Yes ☒ No

Reporting for specific materials, systems and methods

We require information from authors about some types of materials, experimental systems and methods used in many studies. Here, indicate whether each material, system or method listed is relevant to your study. If you are not sure if a list item applies to your research, read the appropriate section before selecting a response.

Materials & experimental systems

n/a	Involved in the study
☒	☐ Antibodies
☒	☐ Eukaryotic cell lines
☒	☐ Palaeontology and archaeology
☐	☒ Animals and other organisms
☒	☐ Clinical data
☒	☐ Dual use research of concern
☒	☐ Plants

Methods

n/a	Involved in the study
☒	☐ ChIP-seq
☒	☐ Flow cytometry
☒	☐ MRI-based neuroimaging

Animals and other research organisms

Policy information about studies involving animals; ARRIVE guidelines recommended for reporting animal research, and Sex and Gender in Research

Laboratory animals	The study did not involve laboratory animals
Wild animals	We exclusively used specimens collected from Zoos by the IZW (Institute for Zoo and Wildlife Research) according to CITES regulations.
Reporting on sex	Sex was not considered in our study design.
Field-collected samples	Study did not involve samples collected from the field.
Ethics oversight	No ethical approval or guidance was required since the study did not involve live animals. This study did not involve samples collected from the field.

Note that full information on the approval of the study protocol must also be provided in the manuscript.

Plants

Seed stocks	n/a
Novel plant genotypes	n/a
Authentication	n/a
